# Detergent-Enzymatic Decellularization of Swine Blood Vessels: Insight on Mechanical Properties for Vascular Tissue Engineering

**DOI:** 10.1155/2013/918753

**Published:** 2013-06-20

**Authors:** Alessandro F. Pellegata, M. Adelaide Asnaghi, Ilaria Stefani, Anna Maestroni, Silvia Maestroni, Tommaso Dominioni, Sandro Zonta, Gianpaolo Zerbini, Sara Mantero

**Affiliations:** ^1^Department of Chemistry, Materials and Chemical Engineering “Giulio Natta”, Politecnico di Milano, Milan, Italy; ^2^PhD program in Bioengineering, Politecnico di Milano, Milan, Italy; ^3^Complication of Diabetes Unit, Division of Metabolic and Cardiovascular Sciences, San Raffaele Scientific Institute, Milan, Italy; ^4^General Surgery I, Fondazione IRCCS Policlinico San Matteo, Pavia, Italy

## Abstract

Small caliber vessels substitutes still remain an unmet clinical need; few autologous substitutes are available, while synthetic grafts show insufficient patency in the long term. Decellularization is the complete removal of all cellular and nuclear matters from a tissue while leaving a preserved extracellular matrix representing a promising tool for the generation of acellular scaffolds for tissue engineering, already used for various tissues with positive outcomes. The aim of this work is to investigate the effect of a detergent-enzymatic decellularization protocol on swine arteries in terms of cell removal, extracellular matrix preservation, and mechanical properties. Furthermore, the effect of storage at −80°C on the mechanical properties of the tissue is evaluated. Swine arteries were harvested, frozen, and decellularized; histological analysis revealed complete cell removal and preserved extracellular matrix. Furthermore, the residual DNA content in decellularized tissues was far low compared to native one. Mechanical testings were performed on native, defrozen, and decellularized tissues; no statistically significant differences were reported for Young's modulus, ultimate stress, compliance, burst pressure, and suture retention strength, while ultimate strain and stress relaxation of decellularized vessels were significantly different from the native ones. Considering the overall results, the process was confirmed to be suitable for the generation of acellular scaffolds for vascular tissue engineering.

## 1. Introduction

Cardiovascular diseases still represent one of the leading causes of death in the world [1]. Current clinical approaches showed a poor efficacy especially in small diameter artery substitutions (<6 mm).  Autologous transplantation using saphenous vein or mammary artery is currently the best solution; however, this approach is not always feasible, since most vessels are often affected by diffuse atherosclerotic abnormalities and only few vessels remain indeed suitable for this purpose. Another option is represented by the use of synthetic grafts, which allow positive outcomes when used in large diameter vessels substitution but often lead to the failure of the implant due to thrombotic phenomena or intimal hyperplasia when applied in small caliber ones [2]. The mechanical behavior of the implant was proven to be one of the main causes of lumen occlusions because of compliance mismatch between the native vessel and the synthetic graft [3, 4]; in order to compensate for the mechanical difference, an in vivo remodeling occurs leading to progressive vessel wall thickening, until complete occlusion. Furthermore, mechanical mismatching could generate nonphysiological blood flows encouraging the formation of thrombi or aneurysms. Based on these observations, the ideal vascular graft should resist the physiological arterial pressure and should avoid any mechanical mismatch. Tissue engineering can overcome the limitations of the currently available vessel substitutes through the generation of biologically based functional vessels which could more closely replicate the physiological tissue. Better tuned biochemical and mechanical properties of tissue-engineered blood vessels (TEBV) can lead to long-term patency even in the case of small caliber conduits. Different approaches have already been investigated in this context and have shown promising results in the last years [5–8].

One technique that has shown good preclinical and clinical results in several tissue engineering applications [9–12], including blood vessels [7], is the use of decellularized scaffolds. Decellularization is the complete removal of all cellular and nuclear matters from a tissue preserving its native extracellular matrix (ECM) and could be performed through physical, chemical, and enzymatic agents [13]. The obtained acellular scaffolds represent a great substrate able to promote cell adhesion, differentiation, and proliferation thanks to the specific ECM components and structure. Moreover, decellularized scaffolds sport mechanical properties similar to those of native tissues, a key feature for blood vessels tissue engineering as discussed earlier.

Following the promising results collected in a clinical scenario in the context of trachea engineering [14, 15], we are exploring the feasibility of transposing that methodology to blood vessels in vitro regeneration. To this aim, the present work focuses on the decellularization of porcine arteries investigating cell removal, extracellular matrix preservation, and mechanical properties of the treated vessels compared to native ones.

In addition to this, analysis and considerations are made regarding the generation of an “off the shelf” product, desirable for an effective translation of tissue-engineered tissues to the clinic. Since explants from cadavers are the main source for the production of decellularized scaffolds, as much tissue as possible would need to be harvested whenever available and the storage of the starting material acquires a key relevance. For this reason, we also analyzed how the −80°C storage affects the mechanical properties of explanted vessels prior to decellularization. 

## 2. Materials and Methods

### 2.1. Tissue Harvesting

Arterial segments (inner diameter from 2 to 11 mm) were obtained from abdominal aorta and carotid from 10 pigs (*Large White Piglets*) under general anesthesia and orotracheal ventilation. The study was carried out under Ethical Committee for Animal Experimentations of University of Pavia (permit 1/2012) approval and following Italian laws for animal care. Fine dissection was performed using scalpel and scissor, providing complete removal of loose connective tissue around the vessels. Each segment was rinsed three times in Dulbecco's phosphate buffered saline (PBS) containing 1% penicillin, 1% streptomycin, and 1% amphotericin B (AA solution; all reagents Sigma-Aldrich, St. Louis, MO, USA). Vessels were either directly processed for characterization of the native tissue or stored dry at −80°C until decellularization

### 2.2. Decellularization

Vessels were decellularized according to a previously tested protocol using an in-house developed device for the automatic decellularization of biological tissues [16]. Briefly, the treatment consists of 4 cycles as follows: 72 h washing in deionized water with 1% AA solution at 4°C followed by 4 h in sodium deoxycholate 4% solution at room temperature (RT) and 3 h in deoxyribonuclease I 2000 kU in sodium chloride 1 M at RT. The whole process was done under agitation and between each step the vessel was rinsed 3 times in PBS (all reagents Sigma-Aldrich, St. Louis, MO, USA).

### 2.3. Histology

At retrieval and after decellularization specimens of the vessels were isolated and processed in paraformaldehyde 4% in PBS, then embedded in paraffin, and 4 *μ*m slices were stained with hematoxylin-eosin and 4′,6-diamidino-2-phenylindole (DAPI) as nuclear marker.

### 2.4. DNA Quantification

At native condition and after decellularization the total amount of DNA was quantified using a DNA isolation kit for tissues (Qiagen, Venlo, The Netherlands). Briefly, 40 mg wet weight samples of native and decellularized arteries were digested using a cell lysis buffer and Proteinase K followed by a protein precipitation solution and centrifugation to remove the protein fraction. In order to isolate the DNA, the supernatant was added with isopropanol and ethanol and then centrifuged and rehydrated with a DNA rehydrating solution. Total DNA was finally quantified using a spectrophotometer at 260 nm.

### 2.5. Scanning Electron Microscopy

Samples of native, defrozen, and decellularized arteries were fixed with 2.5% glutaraldehyde for 1 h and stored in sodium cacodylate 0.1 M. Dehydration was achieved using an ethanol scale and samples were gold coated before being analyzed with a Zeiss EVO 50 environmental scanning electron microscope (ESEM).

### 2.6. Mechanical Testing

Native, defrozen, and decellularized vessels were cut into ring-shaped specimens for mechanical analysis. Native tissue samples were tested within 4 hours of cold ischemia after retrieval. Mechanical testing was performed according to a previously developed protocol [17] with some modifications as follows: 5 mm wide specimens were mounted on custom-made holders on a computer-controlled uniaxial testing machine (Synergie 200 H MTS, Minneapolis, MN, USA), and data were acquired using the Test Work software (MTS, Minneapolis, MN, USA). A preloading of 0.015 N was imposed and the corresponding length was measured. Each sample was preconditioned with loading-unloading cycles until a reproducible response was achieved, with a loading speed of  1.0 mm/min and a load end point of 0.08 N/mm. The sample was left to recover for 120 seconds, repeatedly rehydrated with PBS, and a relaxation test was then performed, with a ramp speed of 0.1 mm/s. The ramp end point was set at 0.08 N/mm. The test was stopped after 500 seconds and the sample left to recover for additional 120 seconds, repeatedly rehydrated with PBS. A failure tensile test was finally carried out with an actuator speed of  1.0 mm/min until complete sample rupture. Measurements of the vessels wall thickness were made using a digital camera Canon EOS 350D (Canon, Tokyo, Japan) equipped with a macro lens to avoid optical distortion. Images were processed using ImageJ software (ImageJ, NIH, USA) measuring the mean inner diameter (ID) and the mean wall thickness (WT). Thirty samples representatives of the 2–11 mm ID range were analyzed and an ID-WT relation was calculated by interpolation of the experimental dataset. The data from the mechanical testing were processed using Open Office Calc (Apache Software Foundation, Forest Hill, USA). From the loading ramp of the relaxation test, the Stiffness (*K*—[N/mm]) was evaluated as

(1) $$\begin{array}{c} K=\frac{T_{0.08}-T_{0.04}}{\varepsilon_{0.08}-\varepsilon_{0.04}}, \end{array}$$

where *T* is the tensile stress (N/mm) and *ε* the circumferential strain (mm/mm). The vessel wall thickness allowed to calculate the circumferential Young modulus (*E*_circ_—[MPa]) derived as

(2) $$\begin{array}{c} E_{\text{circ}}=\frac{K}{2\text{WT}}, \end{array}$$

where WT is the wall thickness derived from the ID-WT relation (see previous paragraph). Compliance was derived from a mathematical model previously described [18], introducing an equivalence criteria between *T* and the internal pressure (*P*), and between *ε* and the diameter (*D*). Moreover, from the relaxation test we evaluated the residual stress (*G*) as the ratio between *T* after 500 s and *T* at the end of the loading ramp:

(3) $$\begin{array}{c} G=\frac{T_{500\;\text{s}}}{T_{0}}. \end{array}$$

From the rupture test we estimated the ultimate tensile stress (*T*_max⁡_—N/mm) and the ultimate strain (*ε*_max⁡_—mm/mm) as the maximum *T* and *ε* values before failure of the sample; like Young's modulus, the circumferential ultimate stress (*σ*_max⁡_—MPa) was calculated using the ID-WT relation as

(4) $$\begin{array}{c} \sigma_{\max}=\frac{T_{\max}}{2\text{WT}}. \end{array}$$

The burst pressure (BP—mmHg) was calculated from the rupture test; using the same equivalence model used for the compliance, the pressure was derived from the ultimate tensile stress and the diameter was derived from the ultimate strain.

The suture retention test was performed according to the standard ISO 7198; vessel segments of 20 mm in length were clamped to the fixed holding of the testing machine, and on the other end of the vessel, a single bite of 5–0 Prolene was placed 2 mm under the edge and pulled at a constant rate of 80 mm/min until failure. For each vessel the test was repeated three times, with each suture bite at 120° with respect to the other. The force needed to pull away the suture was measured and reported as suture retention strength (SR—g).

### 2.7. Statistical Analysis

Statistical analysis was performed using GraphPad Prism (GraphPad Software Inc., La Jolla, CA, USA) and data were compared using a Kruskal-Wallis test with Dunn post-test with significance limit set for *P* < 0.05.

## 3. Results

### 3.1. Histological Results

**Figure 1:**
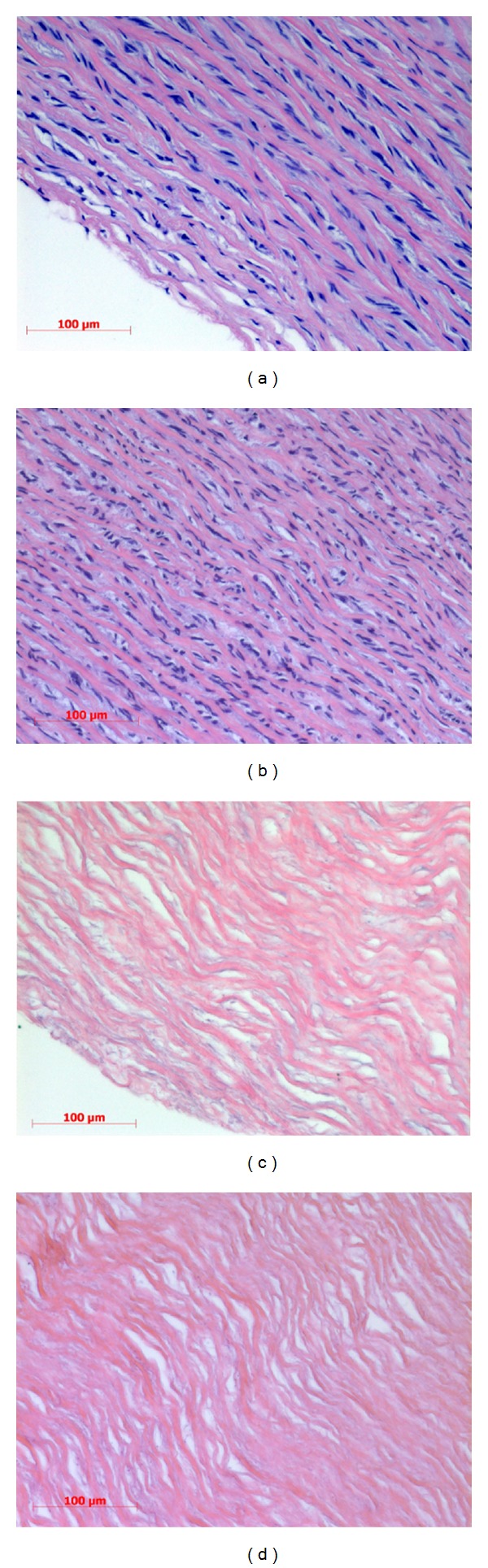
Hematoxylin and eosin staining of swine arteries. Hematoxylin and eosin staining of native ((a)-(b)) and decellularized ((c)-(d)) porcine arteries; the images show a complete absence of nuclei or cellular residues and a preserved ECM structure at the end of the process.

**Figure 2:**
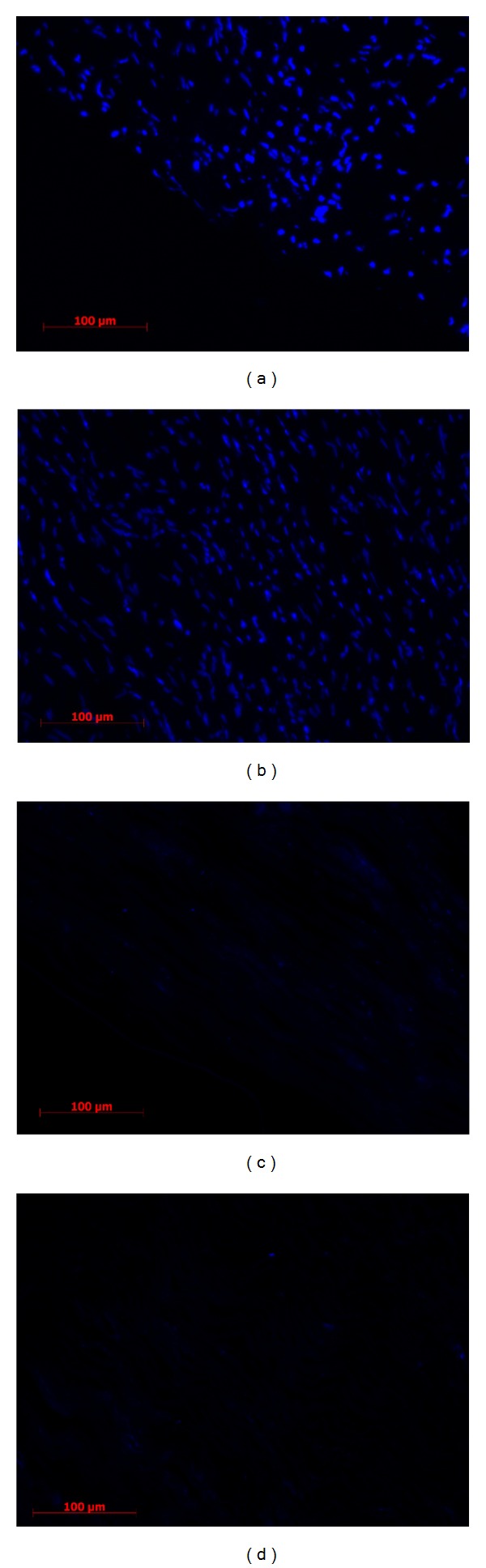
DAPI staining of swine arteries. DAPI fluorescent staining of native ((a)-(b)) and decellularized ((c)-(d)) porcine arteries; the results confirm the absence of nuclei in the decellularized tissue.

Native and decellularized vessel samples were stained with hematoxylin-eosin and DAPI. The overall histological results, as shown in Figures 1 and 2, revealed the absence of cells or nuclear matter. Hematoxylin and eosin staining (Figure 1) showed a preserved ECM structure at the end of the decellularization process with no residual cells, confirmed by DAPI fluorescent staining (Figure 2).

### 3.2. DNA Quantification Results

**Figure 3:**
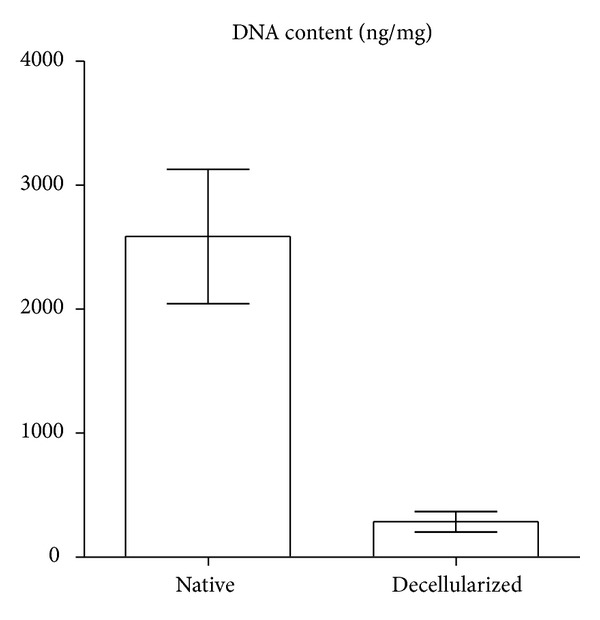
Residual DNA content. DNA content for native and decellularized swine arterial vessels; data are reported as mean ± standard deviations.

The spectrophotometric analysis revealed that the decellularization process removed the majority of the DNA content in the treated tissue compared to the native one (286 ± 83 ng/mg versus 2586 ± 542 ng/mg) (Figure 3).

### 3.3. Scanning Electron Microscopy Results

**Figure 4:**
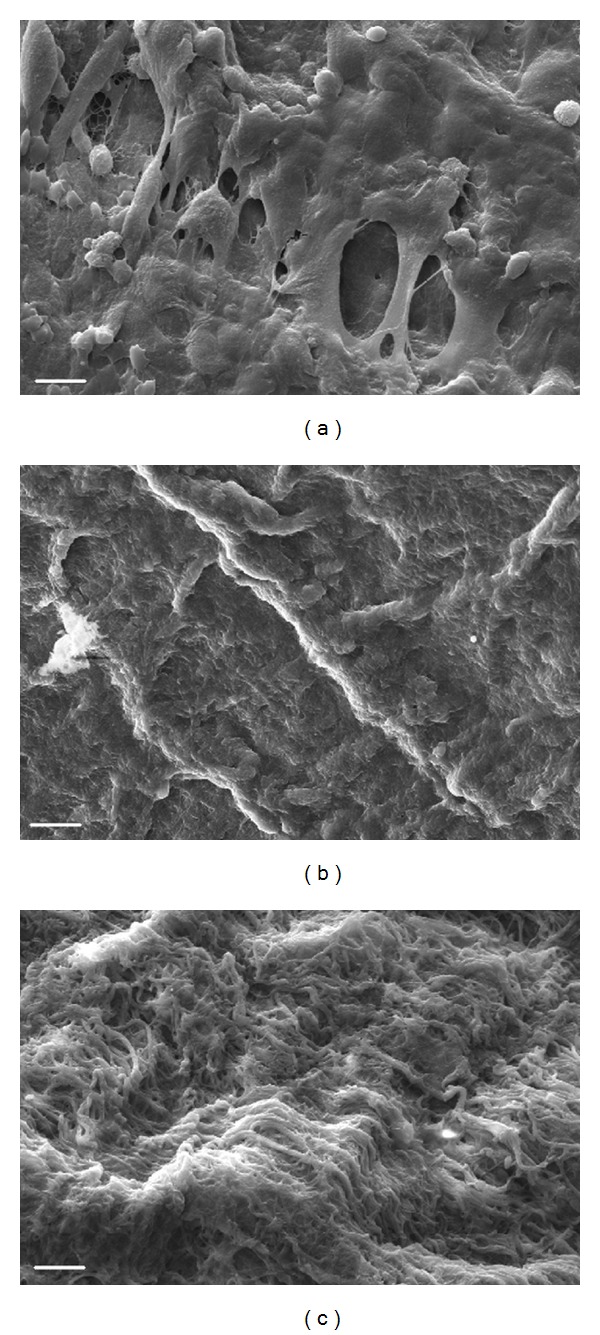
Environmental Scanning Electron Microscopy. ESEM analysis of the inner lumen of native (a), defrozen (b), and decellularized (c) porcine arteries. Bars 10 *μ*m.

Native, defrozen, and decellularized vessels samples were freeze dried, gold coated, and observed with ESEM. The reported images refer to the luminal surface of the vessels (Figure 4); no evidence of damage can be observed after neither the freezing step (Figure 4(b)) nor the decellularization process (Figure 4(c)).

### 3.4. Vessel Inner Diameter-Wall Thickness Relation

**Figure 5:**
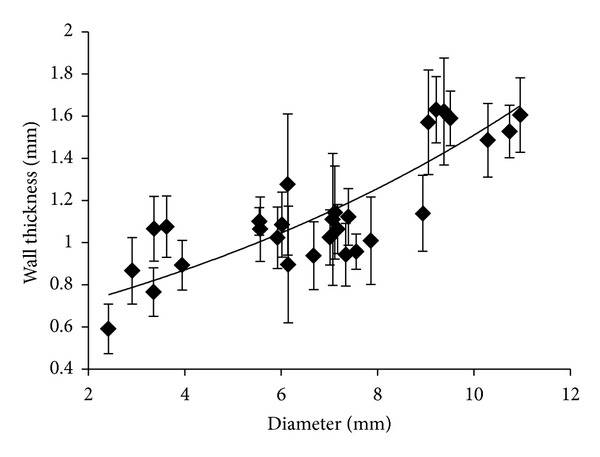
Wall thickness to inner diameter relation. Wall thickness to inner diameter relation derived from optical acquisition of pig arterial samples sections.

In order to calculate circumferential stress and Young's modulus, the area of the vessel wall has to be estimated [17]. While the length of the ring specimen is known (5 mm), the wall thickness was measured by using an optical setup and related to the diameter of the vessel. The results are shown in Figure 5.

The interpolating function is

(5) $$\begin{array}{c} \text{WT}=0.615e^{0.088\text{ID}}, \end{array}$$

where WT is the wall thickness and ID the inner diameter; *R* ^2^-value of the curve is 0.6894.

### 3.5. Mechanical Testing Results

**Figure 6:**
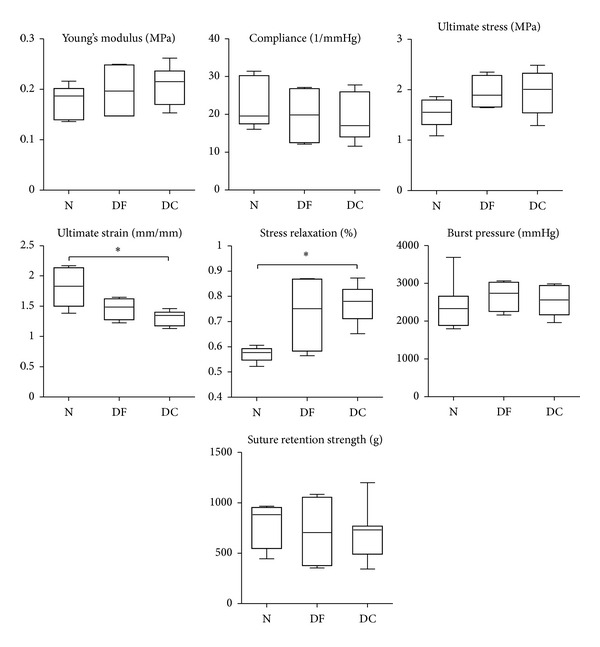
Mechanical analysis. Mechanical testing results for native (N), defrozen (DF), and decellularized (DC) swine arterial vessels. Data are reported as median and 5–95 percentiles, *P < 0.05.

The mechanical testing analysis (Figure 6) resulted in no statistically significant differences for Young's modulus, compliance, ultimate circumferential stress, burst pressure, and suture retention strength; on the other hand, there was a significant loss in ultimate strain between native and decellularized vessels; moreover, residual stress after relaxation was increased for decellularized samples compared to native ones. 

## 4. Discussion

Decellularized scaffolds have been used to substitute various tissues with promising results, reaching clinical application. The extracellular matrix represents a good substrate in terms of biochemical properties able to drive cell adhesion, proliferation, and differentiation [19]; moreover, acellular scaffolds usually show good biomechanical properties. The present work is focused on the decellularization of arterial tissue, translating the process we have previously used in the context of engineered trachea transplantation with positive results in terms of both biochemical and mechanical properties of the treated matrix [14]. Cell removal and mechanical properties of decellularized swine blood vessels are here investigated and compared to the native tissue.

The process showed good results; no residual cells were observed in the hematoxylin-eosin (HE) staining, and this condition was confirmed by DAPI analysis where no lasting nuclei were marked. Furthermore, the HE staining revealed a well-maintained structure of the vessel with preserved and organized laminae.

The residual DNA content after decellularization was far low from the quantity found in the native tissue; by now it is unclear whether this few residual DNA could elicit or not an adverse response from the host [20], and indeed many commercial products with positive clinical outcomes contain remnant DNA and it seems unlikely that residual fragments could play a role in any host response [21].

SEM analysis reported a preserved basal layer on the inner lumen of the vessel without evidence of any damaged area. Having a well-preserved inner layer is very important for tissue remodeling in the vascular field since this avoids the promotion of early thrombi and the molecular signaling of the ECM can promote the re-endothelialization. This last aspect takes even more importance when considering the possibility of naked implants (decellularized scaffold implanted without cell seeding) as suggested by Dahl et al. [7]; investigating whether a prior-to-grafting endothelial seeding is necessary or not becomes intriguing in the presence of a signal-rich surface like the acellular one. A proper characterization of the decellularized lumen, considering the most important proteins related to endothelium (or thrombi) formation, will help in the interpretation of the phenomena.

Considering mechanical properties, Young's modulus, compliance, circumferential ultimate stress, burst pressure, and suture retention strength showed no significant differences between nontreated and decellularized samples, while acellular specimens had a lower ultimate strain and a higher residual stress compared to the native tissue. Among these parameters, the compliance is identified as the most crucial factor in eliciting intimal hyperplasia in case of mismatch at the anastomosis sites [4] and in the vessel response to blood pressure affecting tissue remodeling too. With no statistically significant differences in terms of compliance compared to native tissue samples, the decellularized vessels hold promises of in vivo performance closely resembling the physiological ones. Considering the overall outcome, the decellularization process leads to limited modifications of the treated tissues compared to native ones. The histological analysis reported no modifications in the ultrastructure of the matrix, with no unfolded laminae and a well-preserved compact structure; however, the loss of the cellular component could introduce some alterations in the lamina-to-lamina interactions, which could be responsible for the slight modifications we have observed in the mechanical behavior (adhesiveness between two adjacent layers). Regarding this, various studies reported changes in the mechanical properties of decellularized tissues. However, discrepancies among the reported results exist and the data are hardly comparable because of the different testing methods applied and the diverse parameters investigated. Only few works have evaluated time-dependent mechanical parameters modifications on decellularized scaffolds; Zou and Zhang [22] reported the same trend we have observed for stress relaxation and hypothesized the cause being the absence of smooth muscle cells (SMCs). Williams et al. [23] claimed no differences between native and decellularized arteries; however, the reference native vessels in that study were stored at −20°C prior to characterization, a condition that can alter SMCs functionality. These findings corroborate our results, where no statistically significant differences were found in both native/defrozen and defrozen/decellularized comparisons, while some parameters were significantly different between native and decellularized samples.

The translation of tissue engineering techniques to the clinic setting is currently in the eye of the storm. A crucial factor in the case of decellularized scaffolds is the availability of the starting material at the time of need; since the sources for these acellular scaffolds are cadaveric donors, the tissue availability often does not match with patient needs. Expecting a prior-to-process storage as the most frequent situation, we analyzed the effect of −80°C freezing on fresh arterial tissue. Our results showed that this storage technique does not bring any significant mechanical alteration, confirming and integrating (stress-relaxation tests) previous results from Stemper et al. [24]. In order to properly address the whole decellularized scaffold production for future clinical use, further work will be aimed at investigating suitable storage methods for the decellularized matrices. 

## 5. Conclusions

In the last years, decellularized scaffolds emerged inside the tissue engineering scenario as a promising solution for various tissue; the complete removal of cellular and nuclear matter provides a promising substrate in terms of biochemical and mechanical properties. This method reaches a key importance in the small caliber blood vessels field where few clinical substitutes are available.

The aim of this work was to assess the outcome on swine arteries of a detergent-enzymatic decellularization protocol; in summary, the proposed method led to complete cell removal, preserved ECM, and limited alteration of the mechanical properties of the tissues; moreover, freezing of the starting material was also taken into account and evaluated as a possible storage step in a future clinical scenario.

In conclusion, the present work confirmed decellularization as a promising solution for the production of blood vessel acellular matrices for vascular tissue engineering applications.
