# Erratum to “Detergent-Enzymatic Decellularization of Swine Blood Vessels: Insight on Mechanical Properties for Vascular Tissue Engineering”

**DOI:** 10.1155/2014/412838

**Published:** 2014-01-05

**Authors:** Alessandro F. Pellegata, M. Adelaide Asnaghi, Ilaria Stefani, Anna Maestroni, Silvia Maestroni, Tommaso Dominioni, Sandro Zonta, Gianpaolo Zerbini, Sara Mantero

**Affiliations:** ^1^Department of Chemistry, Materials and Chemical Engineering “Giulio Natta”, Politecnico di Milano, Piazza Leonardo da Vinci, 32 20133 Milano, Italy; ^2^PhD Program in Bioengineering, Politecnico di Milano, Piazza Leonardo da Vinci, 32 20133 Milano, Italy; ^3^Complication of Diabetes Unit, Division of Metabolic and Cardiovascular Sciences, San Raffaele Scientific Institute, Milan, Italy; ^4^General Surgery I, Fondazione IRCCS Pol. San Matteo, Pavia, Italy

In results section, the compliance *y*-axis in Figure  6 had wrong values and now it is correct; furthermore, Table  1 representing median and percentile values of parameters reported in Figure 6 is added.

The correct text for the paragraph is as follows.

*3.5. Mechanical Testing Results*. The mechanical testing analysis (Figure  6, Table  1) resulted in no statistically significant differences for Young's modulus, compliance, ultimate circumferential stress, burst pressure, and suture retention strength; on the other hand, there was a significant loss in ultimate strain between native and decellularized vessels; moreover, residual stress after relaxation was increased for decellularized samples compared to native ones.

## Figures and Tables

**Figure 6:**
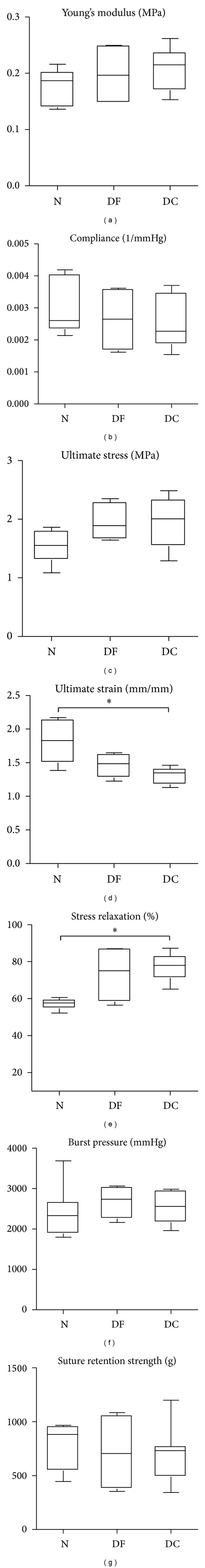
Mechanical analysis. Mechanical testing results for native (N), defrozen (DF), and decellularized (DC) swine arterial vessels. Data are reported as median and 5–95 percentiles, *P < 0.05.

**Table 1:** Median and quartile values of mechanical parameters for native, defrozen, and decellularized arterial vessels.

Mechanical parameters	Native		Defrozen		Decellularized	
	Median	25–75 percentiles	Median	25–75 percentiles	Median	25–75 percentiles
Young's modulus [MPa]	0.1867	0.1396–0.2016	0.1965	0.1473–0.2486	0.2152	0.1699–0.2365
Compliance [1/mmHg]	0.002606	0.002330–0.004033	0.002644	0.001671–0.003574	0.002270	0.001869–0.003454
Ultimate stress [MPa]	1.554	1.309–1.797	1.889	1.658–2.280	2.007	1.540–2.324
Ultimate strain [mm/mm]	1.830	1.499–2.134	1.830	1.274–1.621	1.347	1.174–1.402
Stress relaxation [%]	57.71	54.71–59.23	75.12	58.31–86.88	77.97	71.14–82.79
Burst pressure [mmHg]	2331	1886–2657	2735	2256–3027	2560	2166–2939
Suture retention [g]	881.9	545.2–953.8	705.4	375.8–1055	731.7	490.4–767.3

